# Esthesioneuroblastoma in pediatric and adolescent age. A report from the TREP project in cooperation with the Italian Neuroblastoma and Soft Tissue Sarcoma Committees

**DOI:** 10.1186/1471-2407-12-117

**Published:** 2012-03-25

**Authors:** Gianni Bisogno, Pietro Soloni, Massimo Conte, Marta Podda, Andrea Ferrari, Alberto Garaventa, Roberto Luksch, Giovanni Cecchetto

**Affiliations:** 1 Hematology/Oncology Division, Department of Pediatrics, University, Hospital of Padova, Padova, Italy; 2 Pediatric Hematology/Oncology Division, G. Gaslini Children's Hospital, Genoa, Italy; 3 S.C. Pediatria, Fondazione IRCCS Istituto Nazionale dei Tumori, Milan, Italy; 4 Division of Pediatric Surgery, Department of Pediatrics, University Hospital of Padova, Padova, Italy

**Keywords:** Esthesioneuroblastoma, Olfactory neuroblastoma, Rare tumors, Nasal tumors, Chemotherapy, Radiotherapy, Late effects, Endocrine disorders

## Abstract

**Background:**

Esthesioneuroblastoma (ENB) is a rare, aggressive tumor with no established treatment in children. We analyzed a series of pediatric ENB patients with the aim of improving our knowledge of this disease.

**Methods:**

9 patients (6 males; age 0.9-18 years, median 9.9) were identified by searching the AIEOP (*Italian Association of Pediatric Hematology and Oncology*) registry and the national databases of rare tumors, soft tissue sarcomas (STS) and neuroblastomas. The data on the cases included in STS treatment protocols were collected prospectively and histology was centrally reviewed; the data and histology concerning the other children were reviewed for the purpose of this analysis.

**Results:**

All tumors occurred in the sinonasal region with bone erosion (7 patients) and intracranial (4) or intraorbital (4) extension. Three patients were in Kadish stage B, and 6 in stage C. Complete tumor resection was very difficult to achieve, but adding chemotherapy and radiotherapy enabled tumor control in 8 patients. Response to chemotherapy was evident in 5/7 evaluable cases. Radiotherapy (48.5-60 Gy) was delivered in all children but one, due to early disease progression. With a median follow-up of 13.4 years (range 9.2-22.9), 7 patients are alive in 1^st ^and one in 2nd complete remission. All surviving patients developed treatment-related sequelae, the most frequent being endocrine dysfunctions (4 patients) and craniofacial growth impairments (4 patients).

**Conclusions:**

Our findings confirm that ENB in children has an aggressive presentation, but multimodal therapy can cure most patients. Our results are encouraging but future strategies must optimize treatment in terms of survival and related morbidities.

## Background

Esthesioneuroblastoma (ENB), or olfactory neuroblastoma, is a rare, aggressive tumor of the sinonasal region originating from olfactory neuroepithelium. Its incidence is approximately 0.4-1/1,000,000 population per year and, though it can occur at any age, its incidence peaks in the second and fifth decades of life [1,2]. No gender predilection has been reported and its etiology is unknown, but an infectious genesis has been suggested because the tumor contains viral particles [3,4].

In pediatric age, the estimated incidence of ENB is 0.1/100,000 children up to 15 years of age, but it is the most common cancer of the nasal cavity, accounting for 28% of a series of 47 cases registered in the Surveillance, Epidemiology and End Results (SEER) database from 1973 to 2002 [2,5].

ENB in younger patients seems to have a more aggressive presentation than in adults with a larger proportion of cases with advanced disease.

Treatment decisions are based mainly on experience gained in adults, but implementing local measures such as radical surgery and high-dose radiotherapy pose specific problems in pediatric age.

A national-scale initiative called the TREP project (Tumori Rari in Età Pediatrica, *Rare Tumors in Pediatric Age*) was launched in Italy in 2000 with the aim of improving the clinical management of children with very rare cancers (defined as pediatric solid malignancies with an annual incidence < 2/million and not considered in other clinical trials) and contributing to the related basic research [6]. As part of this scheme, the present study was designed to describe the clinical characteristics, treatment and outcome of ENB patients treated at Italian pediatric oncology centers.

## Methods

All patients under 18 years of age registered by AIEOP centers with a diagnosis of ENB were included in this analysis. The cases were identified by searching the AIEOP hospital-based registry (where all Italian pediatric oncology centers register the cases they diagnose), the TREP project database (from 2000 onwards), and the database managed by the Italian Working Groups on Neuroblastoma and Soft Tissue Sarcoma. Only patients diagnosed from 1980 to December 2008 were considered to allow for an adequate follow-up.

Tumors were defined according to the staging system proposed by Kadish and modified by Morita, as follows: A - tumors confined to the nasal cavity; B - tumors infiltrating the paranasal cavities; C - tumors extending beyond the nasal and paranasal cavities; D - tumors with metastases [7,8]. The disease was also staged according to the TNM system, where T1 means tumors confined to the organ or tissue of origin, and T2 lesions invade contiguous structures; T1 and T2 are further classified as A or B by tumor diameter < or > 5 cm, respectively; N1 means regional lymph node involvement; and M1 the presence of distant metastases.

There were no specific guidelines for treating ENB so children were treated on the basis of the existing literature and, for pragmatic reasons, included in the ongoing Italian protocols for rhabdomyosarcoma (RMS) (which also included soft tissue neuroectodermal tumors) or neuroblastoma (NBL).

Informed consent to the treatment and to data collection and analysis was obtained for all patients according to institutional guidelines at the time of enrolling patients in the protocols.

Response to chemotherapy was defined as follows: complete response (CR) - clinically or histologically confirmed complete disappearance of disease; partial response (PR) - at least a two-thirds reduction in tumor volume; minor response (MR) - reduction greater than one-third but less than two-thirds; no response or stable disease (SD) - less than one-third reduction in tumor volume; progressive disease (PD) - increase in tumor size or detection of new lesions.

The data on the cases included in the protocols for RMS were collected prospectively and their histology was centrally reviewed; the data and histology on the other children were reviewed for the purpose of this analysis. Response to chemotherapy was re-evaluated on the basis of radiological reports in the two cases for whom no radiological findings were available.

The long-term sequelae were only ascertained by contacting the clinical investigators at the various centers; no additional investigations were conducted on possible late effects for the purpose of this study.

## Results

Overall, 11 patients with ENB were registered, but full details were only available for 9 of them (6 males; age 0.9-18 years, median 9.9). The patients' demographic data are shown in Table 1.

**Table 1:** Clinical characteristics of 9 patients with esthesioneuroblastoma

**Pt**.	Sex/age at dgn (years)	Symptoms	Primary site	Tumor extension	Tumor size	TNM	Kadish stage
1	M/4	Seizures	Rhinopharynx	Intracranial, bone erosion, cervical lymph nodes	> 5 cm	T2b, N1, M0	C
2	M/2	Exophthalmos	Nasal cavity, rhinopharynx and ethmoid sinuses	Orbital cavity, submandibular lymph node, bone erosion	> 5 cm	T2b, N1, M0	C
3	F/16	Recurrent epistaxis	Paranasal sinuses	-	> 5 cm	T1b, N0, M0	B
4	M/10	None	Nasal cavity, rhinopharynx and maxillary sinuses	Bone erosion	< 5 cm	T2a, N0, M0	B
5	M/1	Recurrent epistaxis	Nasal cavity, ethmoid sinuses	Intracranial, bone erosion	> 5 cm	T2b, N0, M0	C
6	M/11	Nasal obstruction	Nasal cavity, maxillary and ethmoid sinuses	-	> 5 cm	T2b, N0, M0	B
7	M/5	Cranial nerve palsy	Nasal cavity pterygomandibular, infratemporal fossae	Intracranial, orbital cavity, bone erosion	> 5 cm	T2b, N0, M0	C
8	F/18	Proptosis	Nasal cavity	Intracranial, orbital cavity, retromandibular and laterocervical lymph nodes, bone erosion	> 5 cm	T2b, N1, M0	C
9	F/17	Headache	Maxillary and ethmoid sinuses	Orbital cavity, bone erosion	> 5 cm	T2b, N0, M0	C

Pt: patient; dgn: diagnosis; M: male; F: female.

Symptoms were non-specific and usually involved nasal obstruction, headache and epistaxis. One child had an epileptic episode and revealed a mass in the olfactory region that extended intracranially.

In addition to computed tomography or magnetic resonance imaging, metaiodobenzylguanidine scans were obtained for 4 patients but none of them were positive. The initial diagnosis was NBL in 2 cases and PNET in 1, but was changed to ENB after central review soon afterwards. Nearly all tumors were large (> 5 cm in maximum diameter) and aggressive, with bone erosion (7 patients) and intracranial (4) or intraorbital (4) extension. Regional lymph nodes were involved in 3 children. The Kadish stage was consequently C in 6 patients and B in 3.

### Treatment

The treatments administered are summarized in Table 2. At diagnosis, tumor resection was attempted in 4 cases but was always incomplete, while only a diagnostic biopsy was obtained in 4. Lymph node biopsy was performed in one case. No major postoperative complications were reported. All patients received multidrug chemotherapy: 6 were enrolled in protocols proposed for children with RMS and 3 in protocols for children with NBL. The regimens changed over time but the children on the RMS protocol mainly received chemotherapy based on the association of vincristine, doxorubicin, ifosfamide, actinomycin D (VAdIA), while those on the NBL protocol were given cycles with vincristine, doxorubicin and cyclophosphamide (VAdC) alternated with the cisplatin-etoposide combination. The duration of chemotherapy varied considerably, with a total of 5 to 15 cycles being administered.

**Table 2:** Treatment details for 9 patients with esthesioneuroblastoma

**Pt**.	Protocol type	Initial surgery	CT (No. of cycles)	Response to CT	Delayed surgery	RT (dose)	Outcome (years after diagnosis)	Long-term sequelae
1	RMS	Macroscopic residuals	VAdIA (9)	PR	No	No	DOD (0.7)	-

2	RMS	Biopsy	VAdIA (12)	CR	No	Yes (53 Gy)	NED (11.5)	GH deficit, hypogonadism, hypothyroidism, chronic sinusitis, hypovision and cataract, hearing loss, dental abnormalities, facial bones hypoplasia

3	RMS	Biopsy	VAdIA + Carbo/E (5)	PR	Microscopic residuals	Yes (50 Gy)	NED (13)	Palate deformity

4	RMS	Biopsy	VAdIA (12)	MR	Complete resection	Yes (60 Gy)	NED (14)	Hypothyroidism, xerostomia, oligospermia

5	RMS	Microscopic residuals	VAdIA (9)	NE	No	Yes* (42 Gy)	NED (9.2)	Chronic headache, hypothyroidism, attention-deficit/hyperactivity disorder

6	NBL	Macroscopic residuals	VAdC + CDDP/E (6)	SD	Microscopic residuals	Yes (48 Gy)	NED (11.1)	loss of sense of smell, facial bone hypoplasia, recurrent keratoconjunctivitis, maculopathy

7	NBL	Biopsy	VAdCA + CDDP/E+ i.t. MTX (15)	PR	No	Yes (60 Gy)	NED (23)	Amaurosis, hypothyroidism, GH deficiency, xerostomia, facial bones hypoplasia

8	NBL	Lymph node biopsy	VAdC/CDDP (7)	CR	No	Yes (47 Gy)	NED (20.2)	Peripheral neuropathy

9	RMS	Macroscopic residuals	VAdC (12)	NE	No	Yes (60 Gy)	NED (17.1)	Chronic sinusitis

Pt: patient; CT: chemotherapy; RT: radiotherapy; RMS: rhabdomyosarcoma; V: vincristine, Ad:adriamycin, I: ifosfamide, A actinomycin-D; PR: partial response; DOD: dead of disease; CR: complete response; Gy: grays; NED: not evidence of disease; GH: growth hormone; Carbo: carboplatin; E: etoposide; PR: partial response; MR: minor response; NE: not evaluable; NBL: neuroblastoma; C: cyclophosphamide; CDDP: cisplatin; SD: stable disease; i.t. MTX: intrathecal methotrexate

*pt No. 5 received radiotherapy after tumor relapse. Major tumor shrinkage was evident after chemotherapy in 5 of the 7 cases evaluable (2 CR and 3 PR). Response was not evaluable in 2 patients because the tumor was resected at diagnosis in one (No. 5), and because one child had already been irradiated during initial chemotherapy (No. 9). Delayed tumor resection was attempted in 3 patients and was complete in one. Radiotherapy (47-60 Gy) was delivered during the first-line treatment to all but two children: one progressed just before starting radiotherapy; the other was a very young child who was irradiated only after tumor relapse.

### Outcome

With a median follow-up of 13.4 years (range 9.2-22.9), 7 patients are alive in 1^st ^CR. The young child who was not irradiated during first-line therapy (No. 5) relapsed in the locoregional lymph nodes 20 months after completing the treatment, but achieved a 2^nd ^long-lasting CR after tumor resection and further chemo- and radiotherapy. The disease progressed in one patient (No. 1), who died 9 months after diagnosis. The 5-year progression-free and overall survival (OS) rates were thus 77.8% (36.6%-93.9%) and 88.9% (43.3%-98.4%), respectively.

The most frequent long-term adverse effects were endocrine dysfunctions and craniofacial growth impairment (affecting 4 patients each). Other reported sequelae included ocular damage (2), xerostomia (2), chronic sinusitis (2), damage to permanent teeth (1), loss of the sense of smell (1), and behavioral disorders (1). Fertility problems and neuropathies relating to the chemotherapy administered were also reported.

## Discussion

Our report confirms that ENB is very rare in pediatric age and that its behavior is aggressive, since most children presented with locally disseminated disease. As localized ENB (Kadish stage A) seems to be rare in children, and our series only contained tumors in Kadish stages B and C, our discussion focuses on locally advanced ENB.

Tumor resection is generally the first therapeutic measure in adults with ENB (though this often requires a craniofacial approach), followed by radiotherapy [9]. Chemotherapy is mainly reserved for patients with advanced, recurrent or metastatic disease. Preoperative radiotherapy in doses in the range of 55 to 65 Gy has been preferred by some authors for stage C ENB [10]. These procedures are highly aggressive, however, and skull base surgery is particularly difficult in children because of the small size of the area, and because of the bone and neurovascular structures located in the craniofacial region. Up to one in three patients therefore risk postoperative morbidities, including complications involving the local wounds, the central nervous system (e.g. cerebrospinal fluid leakage and meningitis) and the ocular orbit. Postoperative mortality has also been reported in up to 5% of patients [11]. Radiotherapy may also cause significant late effects in children, including craniofacial growth impairment and damage to the permanent teeth, endocrine dysfunctions, and loss of the sense of smell [12]. In our series, multiple-agent chemotherapy was adopted systematically and judged preferable to invasive surgery as an initial approach. This had numerous advantages for the patients, since no major surgical complications were reported and tumor shrinkage after chemotherapy meant that delayed surgery could be less aggressive. Unfortunately, complete tumor resection was still very difficult to achieve, but our experience suggests that chemotherapy and radiotherapy may be enough to control postoperative residuals, and some patients were cured without any major surgery.

In our experience radiotherapy is the mainstay of treatment for ENB, as it is for other parameningeal pediatric tumors for which surgery cannot be considered oncologically complete. Two young children in our series were not irradiated and both relapsed locally, but one of them was cured thanks to further treatment, including radiotherapy. Experiences and other data in the literature indicate that irradiation should not be withheld, but future studies should address whether a major response to initial chemotherapy might be enough to reduce the burden of irradiation and its likely long-term sequelae. New techniques, such as proton therapy, may also be helpful to limit the side effects of treatment [13,14].

Our findings show that ENB in children can be considered a chemosensitive tumor. This is in agreement with recent reports of tumor size reductions when chemotherapy was given preoperatively [12,15]. The agents most often used in children are doxorubicin, cyclophosphamide/ifosfamide, vincristine, and etoposide, whereas platinum-based regimens are adopted in adults [16].

The above drugs were used in our studies too, and gave rise to a satisfactory response rate. The limited number of children in our series made it impossible to analyze the different regimens separately or make any more precise recommendations on the type and duration of chemotherapy. This could be done by considering other experiences too, comparing and discussing our experience with those of other national groups interested in rare pediatric tumors. This is one of the main future goals of the TREP Project. In our opinion, the overall strategy for unresectable tumors may be similar to the one adopted for other parameningeal tumors, namely RMS, for which intensive chemotherapy and early radiotherapy are recommended.

The survival results reported here are higher than those described in previously-published series. This may be due to the systematic use of a multidisciplinary approach in all the patients concerned. It may also be that ENB is more aggressive in children than in adults and/or more sensitive to current treatments. This seems to be the case for other rare tumors that behave differently in different age groups. Unfortunately, the need for an aggressive treatment approach can also mean severe side effects and this issue should be addressed when planning future treatments.

## Conclusion

In conclusion, our findings confirm that ENB has aggressive features in children, but a multimodal approach - relying mainly on chemotherapy and radiotherapy - can cure most patients. This is an encouraging result, but more data are needed to optimize strategies for treating pediatric ENB in terms of patient survival and treatment-related morbidities.

The limited number of ENB patients analyzed in this collaborative effort as part of the TREP project goes to show that we need to move from national to international cooperative schemes in order to obtain more solid evidence to guide the treatment of such very rare tumors as ENB.

## Competing interests

The authors declare that they have no competing interests.

## Authors' contributions

GB conceived the study, coordinated the data analysis and drafted the manuscript. PS collected the data and cooperated on the data analysis and the drafting of the manuscript. GC reviewed the data on local treatments and critically revised the manuscript. MC, AF, MP, AG, RL made substantial contributions to the conception of the study and to data acquisition, as well as taking part in the final analysis and the drafting of the manuscript. All authors have read and approved the final manuscript.

## Pre-publication history

The pre-publication history for this paper can be accessed here:

http://www.biomedcentral.com/1471-2407/12/117/prepub
